# A systematic review and meta-analyses of the relationship between glutathione S-transferase gene polymorphisms and renal cell carcinoma susceptibility

**DOI:** 10.1186/s12881-018-0620-y

**Published:** 2018-06-08

**Authors:** Zhiqing Zhong, Hongyan Li, Hongzhen Zhong, Tianbiao Zhou, Weiji Xie, Zhijun Lin

**Affiliations:** 1 Department of Nephrology, the Second Affiliated Hospital of Shantou University Medical College, No. 69 Dongsha Road, Shantou, 515041 China; 2 Department of Nephrology, Huadu District People’s Hospital of Guangzhou, Southern Medical University, Guangzhou, 510800 China

**Keywords:** Renal cell carcinoma, *GSTM1*, *GSTT1*, *GSTP1*, Gene polymorphism, Meta-analysis

## Abstract

**Background:**

Association of *GSTM1*- and *GSTT1*-null genotypes, *GSTP1* A/G gene polymorphism with renal cell carcinoma (RCC) susceptibility was detected, and the relationship between the *GSTM1*/*GSTT1*-null genotype and clinical TNM stages of RCC was assessed, using meta-analysis method.

**Methods:**

Association investigations according to eligibility criteria were searched and identified from the databases of Cochrane Library, PubMed, and Embase from establishment time of databases to July 1, 2017, and eligible reports were analyzed by meta-analysis. 95% confidence intervals (CI) were also detected, and odds ratios (OR) was used to express the results for dichotomous data.

**Results:**

This meta-analysis indicated that there was no an association between *GSTM1*-null genotype, *GSTT1*-null genotype, *GSTP1* A/G gene polymorphism and RCC risk in the overall population of Caucasians or Asians. The dual *GSTM1–GSTT1*-null genotype was also not associated with RCC in the overall population of Caucasians. Interestingly, there was an association between the dual *GSTM1-GSTT1*-null genotype and the susceptibility of RCC in Asians. Relationship of the *GSTM1*-null genotype with clinical TNM stage of RCC was not observed in the overall population of Asians or Caucasians. In this meta-analysis, no association between the *GSTT1*-null genotype and clinical TNM stage of RCC was observed in Caucasians or Asians. Interestingly, *GSTT1*-null genotype was detected to be associated with the clinical TNM stages in patients with RCC in the overall population.

**Conclusion:**

The dual *GSTM1-GSTT1*-null genotype is detected to be associated with the onset of RCC in Asians, and there is an association between the *GSTT1*-null genotype and the clinical TNM stages in patients with RCC in the overall population.

**Electronic supplementary material:**

The online version of this article (10.1186/s12881-018-0620-y) contains supplementary material, which is available to authorized users.

## Background

Renal cell carcinoma (RCC) is associated with high mortality, accounts for approximately 80–85% of all renal tumors, and is the most common type of adult kidney cancer with poor prognosis [1]. Approximately 30% RCC patients already have metastatic lesions upon initial diagnosis [2]. Renal cell carcinoma (RCC) is highly resistant to both chemotherapy and radiotherapy [3]. Early diagnosis of patients with RCC would significantly improve their prognosis and quality of life [4–6]. The incidence of survival is very low, since most RCC patients have developed metastases beyond the kidney tissue when the RCC is diagnosed [4, 7, 8]. Early diagnosis for the disease of RCC is very difficult, and the RCC etiology is complicated [4, 8]. Gene polymorphisms are reported to be associated with susceptibility of many diseases [9–13]. Current evidence also shows some gene polymorphisms to be associated with RCC risk [14–17].

The glutathione S-transferases (GSTs) is a family of isozymes including GSTM1, GSTT1, and GSTP1 classes, and can catalyze the glutathione to detoxify xenobiotics [18, 19]. GSTs conjugate glutathione (GSH), a scavenger peptide, with electrophilic compounds [20, 21], and are known to play a pivotal role in the detoxification of some potential carcinogens [22, 23]. It has also been suggested that certain *GST* gene polymorphisms, leading to altered detoxification activity, predispose individuals to certain cancers, such as prostate cancer, hepatocellular carcinoma, and colorectal cancer [24–26].

Previously, most epidemiologic investigations have detected a relationship between the *GSTM1/GSTT*-null phenotype, the *GSTP1* A/G gene polymorphism, and RCC risk. But, the current evidence is inadequate, for the reason that sparseness of data or inconsistencies among these reported investigations. This meta-analysis was conducted to assess whether the null genotype of *GSTM1/GSTT1* and the *GSTP1* A/G gene polymorphism are associated with RCC susceptibility by ethnicity, and whether there is an association between the null genotype of *GSTM1/GSTT1* and clinical TNM stages in patients with RCC by ethnicity, due to the fact that the genotype distributions of the different populations might differ from each other [27, 28]. We also evaluated the publication bias for the relationship between the *GSTM1*-null genotype, *GSTT1*-null genotype, dual *GSTM1/GSTT1*-null genotype, and *GSTP1* A/G gene polymorphism and RCC risk for the overall population.

## Methods

### Search strategy

Retrieval of relevant published articles were conducted in the electronic databases of Cochrane Library, PubMed, and Embase from establishment time of databases to July 1, 2017, and eligible investigations were recruited for our meta-analysis. Key subjects for retrieval consisted of (“glutathione S-transferases” OR “*GSTs*” OR “*GSTM1*” OR “*GSTT1*” OR “*GSTP1*”) and (“renal cell carcinoma” OR “renal cancer” OR “RCC”). Additional reports were also recruited through references which were cited in the included investigations, and references of retrieved articles from previous meta-analyses were also inspected.

### Inclusion criteria and exclusion criteria

#### Inclusion criteria

(1) prospective study, case-control study, and cross-sectional study; (2) there should be two comparison groups (RCC vs. control); (3) the endpoint had to be RCC; (4) the study should provide detailed data for the genotype distribution.

#### Exclusion criteria

(1) primary results were not on *GSTM1*, *GSTT1*, *GSTP1* or outcome; (2) review articles, case reports and editorials; (3) investigated the effect of GST gene expression on disease.

### Quality appraisal

In order to evaluate the quality of the recruited investigations that met the inclusive criteria mentioned above, a quality score criteria based on seven aspects of a genetic association investigations was used (Additional file 1: Table S1). The quality score form was instituted by Thakkinstian et al. in 2005 [29]. Its range of this form spanned from zero (the worst quality) to 12 (the best quality). Investigations were categorized to be “high quality” when the quality score was more than seven; otherwise, studies were regarded as “low quality”. Quality appraisal was implemented by two researchers who were independently responsible for the literature retrieval, and discussions were held until every aspect was entirely consistent by comparison.

### Data extraction and data synthesis

The following information from each eligible study was excerpted by two investigators independently: the surname of first author, publication year and the sample size of RCC cases and controls for *GSTM1*, *GSTT1*, and *GSTP1* genotypes. Frequencies of genotypes for *GSTM1*, *GSTT1* and *GSTP1* were calculated for each case group and control group. The results were compared, and discussion was performed when there was disagreement. Consistency of data extracted by the two researchers was tested and any disagreement was resolved through discussion.

### Statistical analysis

All statistical analyses were performed using Cochrane Review Manager Version 5.3 (Cochrane Library, UK). Fixed-effect model (Mantel-Haenszel method) was used to estimate the pooled statistic. The heterogeneity among the included studies was detected using *I*^*2*^. On the other hand, when the *P-*value from the heterogeneity test was less than 0.1, a random effects model (DerSimonian-Laird method) was conducted. Odds ratios (OR) were used for results of dichotomous data, and 95% confidence intervals (CI) were also counted. A *P* < 0.05 was regarded as statistical significance for the pooled OR. Publication bias was graphically judged from the Begg adjusted rank correlation test [30] and the Egger regression asymmetry test [31], when the number of the included studies was more than six.

## Results

### Study characteristics

**Fig. 1:**
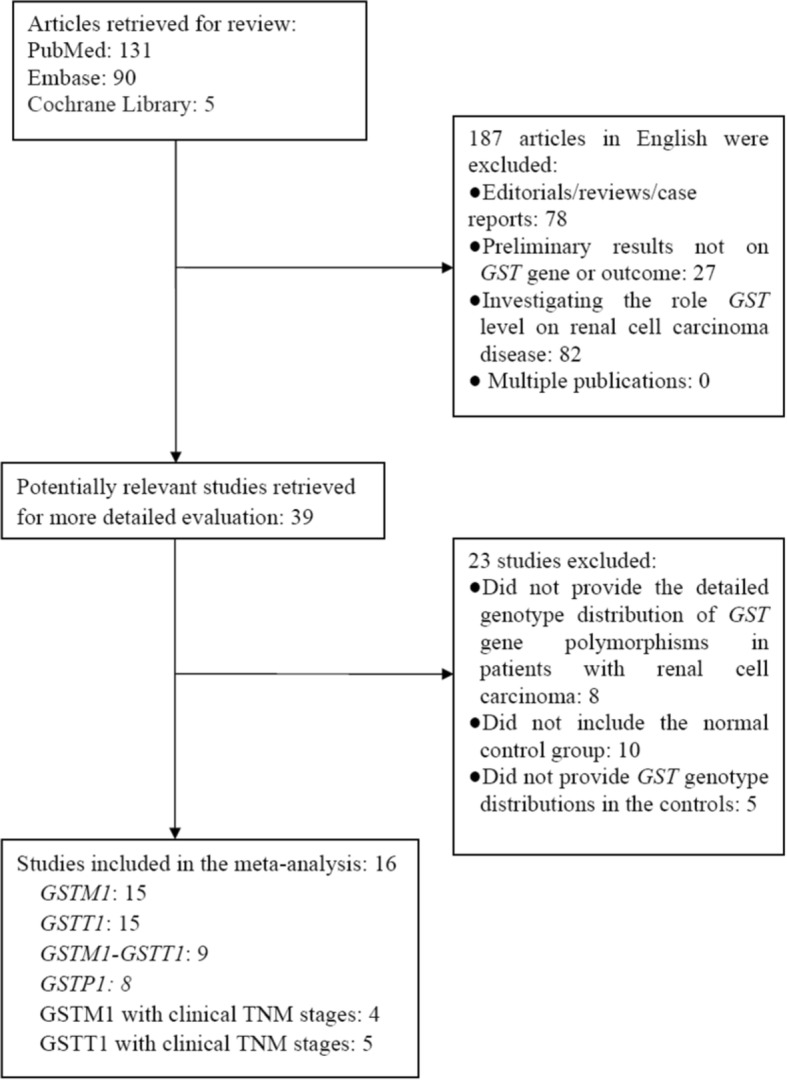
Flow chart of the study search and selection

Fifteen investigations [32–46] were recruited into our meta-analysis to assess the association between the *GSTM1*-null genotype and the susceptibility of RCC (Fig. 1 and Table 1). Data was extracted by the sequences of the surname of first author, publication year and the sample size of RCC cases and controls for the *GSTM1* genotype (Table 1). The 15 included reports contained 3782 cases and 5223 controls. The average *GSTM1*-null genotype distribution frequency in controls was 49.83%, and the average genotype distribution frequency of the *GSTM1-*null genotype in patients with RCC was 48.63%, indicating the average *GSTM1*-null genotype distribution frequency in RCC patients was similar to that in the control group (control/RCC = 1.02), suggesting that the *GSTM1*-null genotype was unrelated to RCC.

**Table 1:** Characteristics of studies evaluating the effects of GSTM1 and GSTT1 null genotypes on RCC risk

Gene	Author, Year	Country	Ethnicity	Source of controls	Quality	Case			Control		
Locus					Score	–	+	Total	–	+	Total
GSTM1	Bruning 1997	Germany	Caucasian	Population-based	6	18	27	45	31	17	48
	Longuemaux 1999	France	Caucasian	Hospital-based	8	89	84	173	117	94	211
	Sweeney 2000	USA	Mix	Population-based	9	63	63	126	255	250	505
	Buzio 2003	Italy	Caucasian	Hospital-based	8	50	50	100	108	92	200
	Moore 2007	Europe	Caucasian	Hospital-based	9	424	487	911	555	677	1232
	Wiesenhütter 2007	Germany	Caucasian	Hospital-based	8	51	47	98	167	157	324
	Karami 2008	Europe	Caucasian	Hospital-based	9	303	321	624	433	454	887
	Coric 2010	Serbia	Caucasian	Hospital-based	8	46	30	76	86	96	182
	De Martino 2010	Austria	Caucasian	Hospital-based	8	80	67	147	59	53	112
	Ahmad 2012	India	Asian	Population-based	11	102	94	196	116	134	250
	Salinas-Sanchez 2012	Spain	Caucasian	Hospital-based	6	57	76	133	78	115	193
	Jia 2014	China	Asian	Population-based	NC	22	28	50	30	30	60
	Coric 2016	Serbia	Caucasian	Hospital-based	8	87	109	196	137	137	274
	Abid 2016	Pakistan	Asian	Hospital-based	8	224	378	602	171	248	419
	Coric 2017	Serbia	Caucasian	Hospital-based	8	169	136	305	163	163	326
GSTT1	Bruning 1997	Germany	Caucasian	Population-based	6	3	42	45	11	37	48
	Longuemaux 1999	France	Caucasian	Hospital-based	8	25	148	173	40	171	211
	Sweeney 2000	USA	Mix	Population-based	9	36	90	126	93	412	505
	Buzio 2003	Italy	Caucasian	Hospital-based	8	11	89	100	35	165	200
	Moore 2007	Europe	Caucasian	Hospital-based	9	167	744	911	209	1023	1232
	Wiesenhütter 2007	Germany	Caucasian	Hospital-based	8	19	79	98	59	265	324
	Karami 2008	Europe	Caucasian	Hospital-based	9	129	499	628	161	752	913
	Coric 2010	Serbia	Caucasian	Hospital-based	8	21	55	76	52	130	182
	De Martino 2010	Austria	Caucasian	Hospital-based	8	27	120	147	23	89	112
	Salinas-Sanchez 2012	Spain	Caucasian	Hospital-based	6	22	110	132	25	138	163
	Ahmad 2012	India	Asian	Population-based	11	125	71	196	106	144	250
	Jia 2014	China	Asian	Population-based	NC	30	18	48	25	35	60
	Coric 2016	Serbia	Caucasian	Hospital-based	8	44	152	196	71	203	274
	Abid 2016	Pakistan	Asian	Hospital-based	8	72	482	554	49	330	379
	Coric 2017	Serbia	Caucasian	Hospital-based	8	79	226	305	89	237	326
GSTM1-GSTT1	Bruning 1997	Germany	Caucasian	Population-based	6	1	44	45	6	42	48
	Sweeney 2000	USA	Mix	Population-based	9	17	109	126	49	456	505
	Moore 2007	Europe	Caucasian	Hospital-based	9	82	829	911	99	1133	1232
	Karami 2008	Europe	Caucasian	Hospital-based	9	363	260	623	508	372	880
	Salinas-Sanchez 2012	Spain	Caucasian	Hospital-based	6	7	126	133	8	185	193
	Ahmad 2012	India	Asian	Population-based	11	71	125	196	54	196	250
	Jia 2014	China	Asian	Population-based	NC	14	34	48	10	50	60
	Coric 2016	Serbia	Caucasian	Hospital-based	8	24	20	44	36	35	71
	Abid 2016	Pakistan	Asian	Hospital-based	8	29	524	553	17	333	350

*NC* not clear

Fifteen studies [32–46] were recruited into our meta-analysis to detect the association of the *GSTT1*-null genotype with RCC susceptibility (Fig. 1 and Table 1). Those 15 investigations contained 3735 cases and 5179 controls. The average *GSTT1*-null genotype distribution frequency in controls was 23.02%and the average *GSTT1*-null genotype distribution frequency in RCC cases was 24.62%. Therefore, the average distribution frequency of the *GSTT1*-null genotype in control group was similar to that in cases (control/RCC = 0.94), suggesting that the *GSTT1*-null genotype was also unrelated to RCC.

Nine studies [32, 34, 36, 38, 41–45] were recruited into our meta-analysis to assess the relationship of the dual-null genotype, of individuals lacking both *GSTM1* and *GSTT1*, and the susceptibility of RCC (Fig. 1 and Table 1). The nine investigations contained 2679 cases and 3589 controls. The average *GSTM1–GSTT1* dual-null genotype distribution frequency in cases with RCC was 23.71% compared to the average frequency of 20.66% in the controls. The average dual-null genotype of *GSTM1–GSTT1* distribution frequency in RCC patients was slightly increased when compared with that in control group (RCC/control = 1.15).

Eight studies [33, 34, 36, 37, 41, 44, 46, 47] were included in our study to detect the association of the null genotype of *GSTP1* with the susceptibility of RCC (Fig. 1 and Table 2). These 8 investigations contained 2197 cases and 3323 controls. The average A allele distribution frequency in controls was 70.44%, and the average A allele distribution frequency in RCC cases was 69.11%. The average A allele distribution frequency of *GSTP1* in control group was similar when compared with that in the RCC group (control/RCC = 1.02), suggesting a lack of association of the *GSTP1* A allele with RCC.

**Table 2:** Characteristics of studies evaluating the effects of GSTP1 gene polymorphism on RCC risk

Author, Year	Country	Ethnicity	Source of controls	Quality Score	Case				Control			
					AA	AG	GG	Total	AA	AG	GG	Total
Longuemaux 1999	France	Caucasian	Hospital-based	8	71	67	22	160	93	75	21	189
Sweeney 2000	USA	Mix	Population-based	9	58	56	16	130	213	216	62	491
Wiesenhütter 2007	Germany	Caucasian	Hospital-based	8	49	43	7	99	134	144	47	325
Moore 2007	Europe	Caucasian	Hospital-based	9	425	390	95	910	577	548	107	1232
Wang 2011	China	Asian	Hospital-based	9	143	55	9	207	173	54	9	236
Ahmad 2012	India	Asian	Population-based	11	71	99	26	196	126	103	21	250
Coric 2016	Serbia	Caucasian	Hospital-based	8	44	–	–	194	115	–	–	274
Coric 2017	Serbia	Caucasian	Hospital-based	8	74	–	–	301	141	–	–	326

Four studies [34, 40, 41, 45] were included in our meta-analysis to detect the relationship of *GSTM1* with clinical TNM stage of RCC (Fig. 1 and Table 3). Those four investigations contained 501 cases and 423 controls. The average *GSTM1*-null genotype distribution frequency in stage I + II was 47.33%, and the average *GSTM1*-null genotype distribution frequency in stage III + IV was 55.76%. The average *GSTM1*-null genotype distribution frequency in stage I + II was slightly reduced than that in stage III + IV (I + II/III + IV = 0.85).

**Table 3:** Characteristics of studies evaluating the effects of GSTM1 and GSTT1 null genotypes on clinical TNM stages of RCC

Gene	Author, Year	Country	Ethnicity	Source of controls	Quality	Stage I + II			Stage III + IV		
Locus					Score	–	+	Total	–	+	Total
GSTM1-TNM	Sweeney 2000	USA	Mix	Population-based	9	50	55	105	15	8	23
	De Martino 2010	Austria	Caucasian	Hospital-based	8	45	29	74	35	38	73
	Ahmad 2012	India	Asian	Population-based	11	53	77	130	49	17	66
	Abid 2016	Pakistan	Asian	Hospital-based	8	77	115	192	93	168	261
GSTT1-TNM	Sweeney 2000	USA	Mix	Population-based	9	29	76	105	6	17	23
	De Martino 2010	Austria	Caucasian	Hospital-based	8	12	62	74	15	58	73
	Ahmad 2012	India	Asian	Population-based	11	72	58	130	53	13	66
	Salinas-Sanchez 2012	Spain	Caucasian	Hospital-based	6	39	40	79	25	11	36
	Abid 2016	Pakistan	Asian	Hospital-based	8	21	161	182	36	200	236

Five studies [34, 40–42, 45] were recruited into this meta-analysis to assess the association between *GSTT1* and clinical TNM stages of RCC (Fig. 1 and Table 3). Those five studies contained 570 cases and 434 controls. The average *GSTT1*-null genotype distribution frequency in stage I + II was 37.15%, compared to the average frequency of 49.1% in stage III + IV patients. The average *GSTT1*-null genotype distribution frequency in stage I + II was notably reduced than the average *GSTT1*-null genotype distribution frequency in stage III + IV (I + II/III + IV = 0.76).

#### Relationship between the *GSTM1*-null genotype and the susceptibility of RCC

**Fig. 2:**
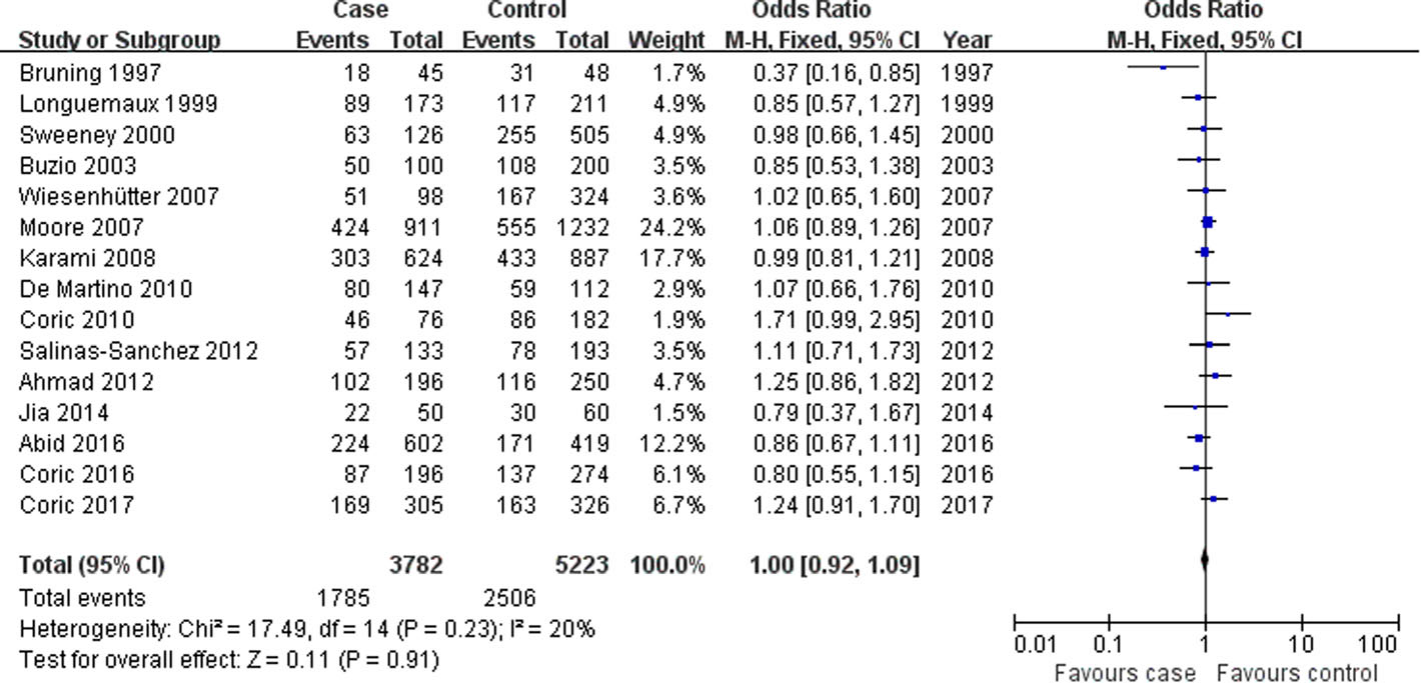
Association between GSTM1 null genotype and RCC susceptibility in the overall population. CI: confidence interval

The *GSTM1*-null genotype was found to be not associated with RCC susceptibility in the collective populations, Asians and Caucasians, hospital-based controls, or population-based controls (collective populations: OR = 1.00, 95% CI: 0.92–1.09, *P* = 0.91; Caucasians: OR = 1.02, 95% CI: 0.92–1.12, *P* = 0.72; Asians: OR = 0.95, 95% CI: 0.78–1.17, *P* = 0.65; hospital-based controls: OR = 1.01, 95% CI: 0.92–1.11, *P* = 0.85; population-based controls: OR = 0.87, 95% CI: 0.57–1.33, *P* = 0.52; Fig. 2 for the overall population; Table 4). When only the high-quality investigations were recruited for meta-analysis, this association was also not found (OR = 1.02, 95% CI: 0.93–1.11, *P* = 0.72; Table 4).

**Table 4:** Meta-analysis of the association of GSTM1- and GSTT1-null genotypes and GSTP1 with RCC risk and the relationship between GSTM1, GSTT1 and clinical TNM stages of RCC

Genetic contrasts	Group and subgroups	Studies Number	Q test *P*-value	Model selected	OR (95%CI)	P
GSTM1						
- vs +	Overall	15	0.23	Fixed	1.00 (0.92,1.09)	0.91
	Caucasian	11	0.16	Fixed	1.02 (0.92,1.12)	0.72
	Asian	3	0.23	Fixed	0.95 (0.78,1.17)	0.65
	Hospital-based	11	0.43	Fixed	1.01 (0.92,1.11)	0.85
	Population-based	4	0.06	Random	0.87 (0.57,1.33)	0.52
	High quality	12	0.42	Fixed	1.02 (0.93,1.11)	0.72
GSTT1						
- vs +	Overall	15	0.0006	Random	1.09 (0.90,1.33)	0.38
	Caucasian	11	0.30	Fixed	1.00 (0.88,1.13)	0.97
	Asian	3	0.005	Random	1.73 (0.91,3.28)	0.09
	Hospital-based	11	0.68	Fixed	1.01 (0.90,1.14)	0.84
	Population-based	4	0.01	Random	1.62 (0.90,2.91)	0.11
	High quality	12	0.002	Random	1.09 (0.90,1.32)	0.39
Dual-null genotype for GSTM1/GSTT1						
- vs +	Overall	9	0.08	Random	1.26 (1.00,1.59)	0.05
	Caucasian	5	0.48	Fixed	1.05 (0.89,1.23)	0.58
	Asian	3	0.22	Fixed	1.72 (1.24,2.38)	0.001
	Hospital-based	5	0.97	Fixed	1.07 (0.91,1.25)	0.43
	Population-based	4	0.12	Fixed	1.70 (1.25,2.32)	0.0007
	High quality	6	0.10	Fixed	1.17 (1.01,1.36)	0.03
GSTP1						
A vs G	Overall	6	0.02	Random	0.93 (0.77,1.11)	0.41
	Caucasian	3	0.06	Random	1.02 (0.80,1.31)	0.85
	Asian	2	0.27	Fixed	0.72 (0.58,0.90)	0.003
	Hospital-based	4	0.10	Fixed	0.97 (0.87,1.08)	0.59
	Population-based	2	0.02	Random	0.82 (0.52,1.29)	0.39
	High quality	6	0.02	Random	0.93 (0.77,1.11)	0.41
AA vs AG + GG	Overall	8	<0.00001	Random	0.74 (0.55,1.00)	0.05
	Caucasian	5	<0.00001	Random	0.72 (0.46,1.13)	0.15
	Asian	2	0.19	Fixed	0.66 (0.50,0.88)	0.004
	Hospital-based	6	<0.00001	Random	0.74 (0.51,1.07)	0.11
	Population-based	2	0.02	Random	0.77 (0.41,1.42)	0.40
	High quality	8	<0.00001	Random	0.74 (0.55,1.00)	0.05
GG vs AG + AA	Overall	6	0.22	Fixed	1.14 (0.93,1.40)	0.22
	Caucasian	3	0.07	Random	0.98 (0.58,1.66)	0.95
	Asian	2	0.51	Fixed	1.49 (0.90,2.49)	0.12
	Hospital-based	4	0.16	Fixed	1.10 (0.87,1.40)	0.43
	Population-based	2	0.21	Fixed	1.26 (0.83,1.91)	0.28
	High quality	6	0.22	Fixed	1.14 (0.93,1.40)	0.22
GSTM1-TNM						
- vs +	Overall	4	<0.0001	Random	0.72 (0.30,1.70)	0.45
	Caucasian	1	–	Fixed	1.68 (0.88,3.24)	0.12
	Asian	2	<0.0001	Random	0.55 (0.11,2.70)	0.46
	Hospital-based	2	0.39	Fixed	1.32 (0.95,1.83)	0.10
	Population-based	2	0.23	Fixed	0.30 (0.18,0.51)	<0.0001
	High quality	4	<0.0001	Random	0.72 (0.30,1.70)	0.45
GSTT1-TNM						
- vs +	Overall	5	0.19	Fixed	0.56 (0.41,0.78)	0.0006
	Caucasian	2	0.36	Fixed	0.56 (0.31,1.01)	0.06
	Asian	2	0.06	Random	0.48 (0.21,1.12)	0.09
	Hospital-based	3	0.55	Fixed	0.64 (0.42,0.97)	0.03
	Population-based	2	0.05	Random	0.54 (0.16,1.87)	0.33
	High quality	4	0.13	Fixed	0.59 (0.41,0.85)	0.004

#### Relationship between the *GSTT1*-null genotype and the susceptibility of RCC

**Fig. 3:**
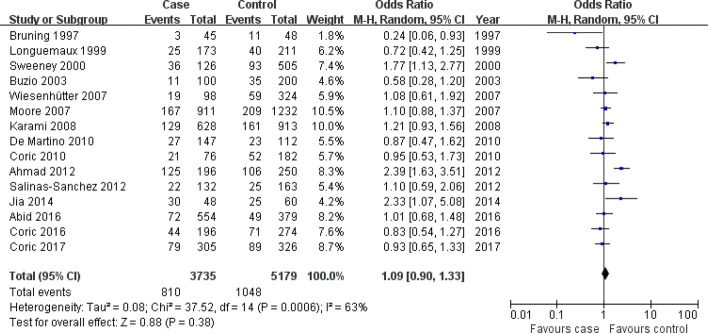
Association between the GSTT1-null genotype and RCC susceptibility in the overall population. CI: confidence interval

Association of *GSTT1* null genotype with RCC risk was not found in the overall population, Caucasians and Asians, hospital-based controls, population-based controls (overall population: OR = 1.09, 95% CI: 0.90–1.33, *P* = 0.38; Caucasians: OR = 1.00, 95% CI: 0.88–1.13, *P* = 0.97; Asians: OR = 1.73, 95% CI: 0.95–3.28, *P* = 0.09; hospital-based controls: OR = 1.01, 95% CI: 0.90–1.14, *P* = 0.84; population-based controls: OR = 1.62, 95% CI: 0.90–2.91, *P* = 0.11; Fig. 3 for the overall population; Table 4). When only the high-quality investigations were included for meta-analysis, an association was also not found (OR = 1.09, 95% CI: 0.90–1.32, *P* = 0.39; Table 4).

#### Association of the dual *GSTM1–GSTT1*-null genotype with the susceptibility of RCC

**Fig. 4:**
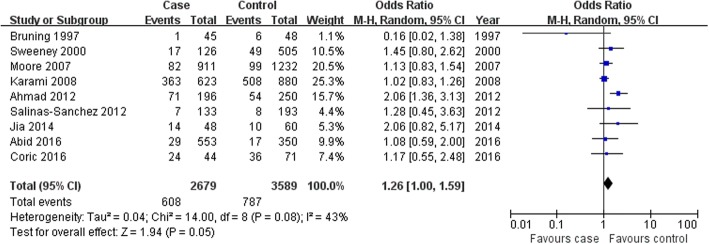
Association between dual-null genotype of GSTM1–GSTT1 with RCC risk in the overall population. CI: confidence interval

There was no an association between the dual-null genotype of individuals lacking both *GSTM1*– and *GSTT1* and RCC risk in the overall population, Caucasians, or hospital-based controls (overall population: OR = 1.26, 95% CI: 1.00–1.59, *P* = 0.05; Caucasians: OR = 1.05, 95% CI: 0.89–1.23, *P* = 0.58; hospital-based controls: OR = 1.07, 95% CI: 0.91–1.25, *P* = 0.43; Fig. 4 for the overall population; Table 4). When only the high-quality studies were recruited for meta-analysis, this association was also not found (OR = 1.17, 95% CI: 1.01–1.36, *P* = 0.03; Table 4). However, stratification into Caucasians and Asians revealed that the dual *GSTM1-GSTT1*-null genotype was associated with the onset of RCC in Asians, when compared to population-based controls (Asians: OR = 1.72, 95% CI: 1.24–2.38, *P* = 0.001; population-based controls: OR = 1.70, 95% CI: 1.25–2.32, *P* = 0.0007; Table 4).

#### Association between the *GSTP1* a/G gene polymorphism and RCC susceptibility

The *GSTP1* A/G gene polymorphism was not associated with RCC risk in the overall population, Asians and Caucasians, hospital-based controls, or population-based controls (overall population: A allele: OR = 0.93, 95% CI: 0.77–1.11, *P* = 0.41; AA genotype: OR = 0.74, 95% CI: 0.55–1.00, *P* = 0.05; GG genotype: OR = 1.14, 95% CI: 0.93–1.14, *P* = 0.22; Table 4). When only the high-quality studies were recruited for the meta-analysis, this relationship was also not found (A allele: OR = 0.93, 95% CI: 0.77–1.11, *P* = 0.41; AA genotype: OR = 0.74, 95% CI: 0.55–1.00, *P* = 0.05; GG genotype: OR = 1.14, 95% CI: 0.93–1.14, *P* = 0.22; Table 4).

#### Relationship between the *GSTM1*-null genotype and clinical TNM stages of RCC

*GSTM1*-null genotype was not associated with the clinical TNM stages of RCC in the overall population, Caucasians, Asians, or hospital-based controls (overall population: OR = 0.72, 95% CI: 0.30–1.70, *P* = 0.45; Caucasians: OR = 1.68, 95% CI: 0.88–3.24, *P* = 0.12; Asians: OR = 0.55, 95% CI: 0.11–2.70, *P* = 0.46; hospital-based controls: OR = 1.32, 95% CI: 0.95–1.83, *P* = 0.10; Table 4). When only the high-quality studies were recruited for meta-analysis, this association was also not found (OR = 0.72, 95% CI: 0.30–1.70, *P* = 0.45; Table 4). Interestingly, the *GSTM1*-null genotype was associated with the clinical TNM stages of RCC when the meta-analysis was compared to population-based controls (OR = 0.30, 95% CI: 0.18–0.51, *P*<0.0001; Table 4).

#### Association of the *GSTT1*-null genotype with clinical TNM stages in patients with RCC

The *GSTT1*-null genotype was not associated with clinical TNM stage of RCC in Caucasians or Asians vs. population-based controls (Caucasians: OR = 0.56, 95% CI: 0.31–1.01, *P* = 0.06; Asians: OR = 0.48, 95% CI: 0.21–1.12, *P* = 0.09; population-based controls: OR = 0.54, 95% CI: 0.16–1.87, *P* = 0.33; Table 4). When only high-quality studies were included for the meta-analysis, association of the *GSTT1*-null genotype with clinical TNM stage of RCC was found (OR = 0.59, 95% CI: 0.41–0.85, *P* = 0.004; Table 4). Interestingly, the *GSTT1*-null genotype was found to be associated with the clinical TNM stages in patients with RCC in the overall population, and when the meta-analysis included hospital-based controls (overall populations: OR = 0.56, 95% CI: 0.41–0.78, *P* = 0.0006; hospital-based controls: OR = 0.64, 95% CI: 0.42–0.97, *P* = 0.03; Table 4).

### Evaluation of publication bias

**Fig. 5:**
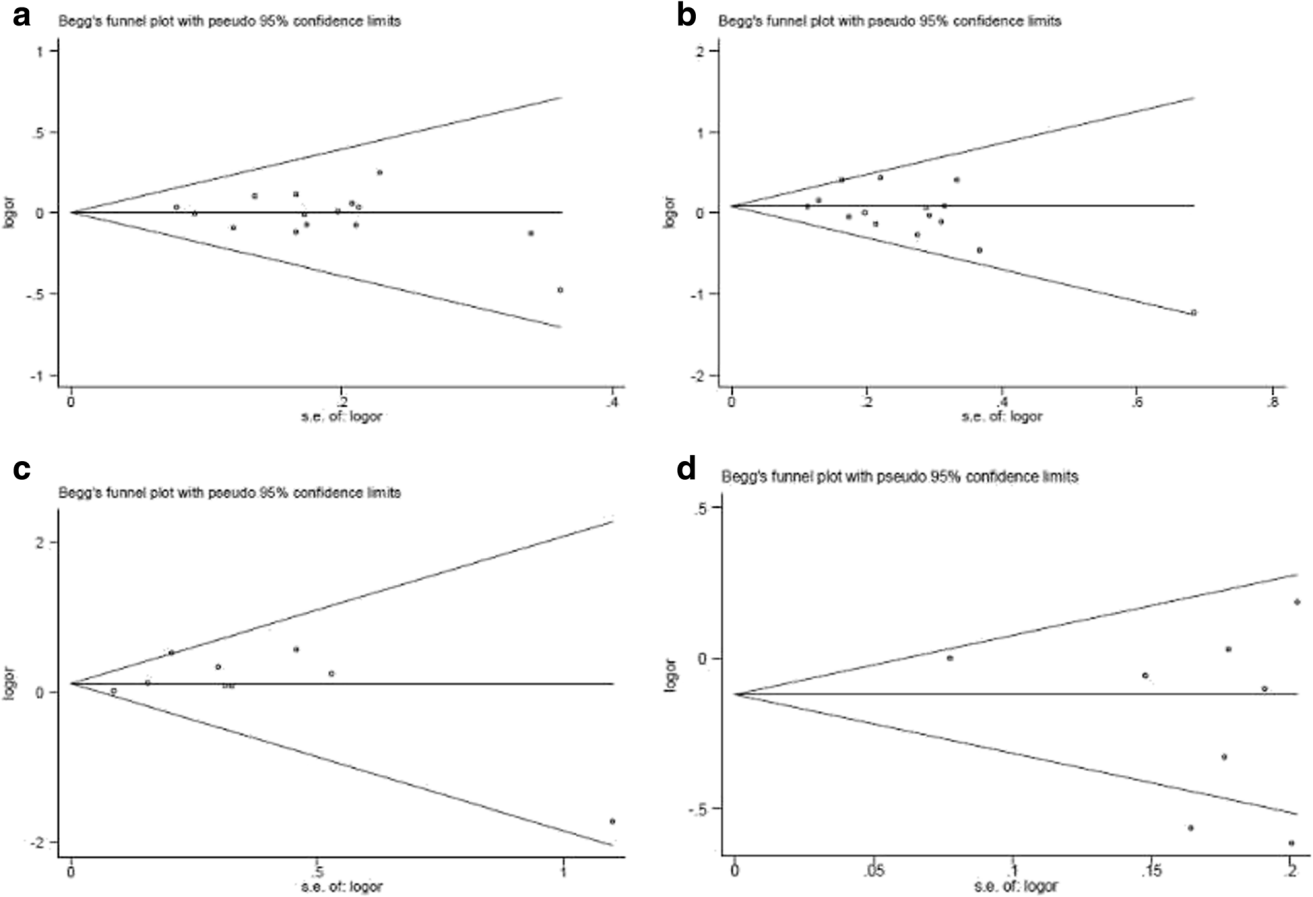
Publication bias A: GSTM1-null genotype; B: GSTT1-null genotype; C: dual null genotype for GSTM1/GSTT1; D: GSTP1 A/G gene polymorphism. Each point represents a separate study for the indicated association. Log or represents natural logarithm of OR. Vertical line represents the mean effects size

A publication bias test was performed for the association of the *GSTM1*-null genotype, *GSTT1*-null genotype, *GSTM1*-null/*GSTT1*-null genotype, and *GSTP1* A/G gene polymorphism with RCC risk, when compared to the overall population. No publication biases for the relationship between the *GSTM1*-null genotype or *GSTT1*-null genotype and RCC risk was determined in the overall population (*GSTM1*: Begg *P* = 0.692, Egger *P* = 0.400; *GSTT1*: Begg *P* = 0.166, Egger *P* = 0.095; *GSTM1*-null/*GSTT1*-null genotype: Begg *P* = 0.917, Egger *P* = 0.628; *GSTP1* A/G gene polymorphism: Begg *P* = 0.902, Egger *P* = 0.290; Fig. 5).

## Discussion

In this study, we found that the average *GSTM1*-null genotype distribution frequency in patients with RCC is similar with the average *GSTM1*-null genotype distribution frequency in the control group, indicating that the *GSTM1-*null genotype is not associated with RCC susceptibility. We performed the meta-analysis in further depth, and still found that there is no an association between null genotype for *GSTM1* and RCC risk in the overall population of Caucasians and Asians, hospital-based controls, population-based controls, high-quality studies. Publication bias was also tested and not found for *GSTM1*. Our results indicate that the *GSTM1*-null genotype does not predict the susceptibility of RCC. The sample size in our meta-analysis was larger than other meta-analyses [43, 48–51].

The average *GSTT1*-null genotype distribution frequency in patients with RCC was also similar to the average *GSTT1*-null genotype distribution frequency in the control group, indicating that the null genotype for *GSTM1* is also not associated with RCC susceptibility. For confirmation, a meta-analysis was performed and showed that there was no an association between null genotype of *GSTM1* and the RCC susceptibility in the overall population, Caucasians and Asians, hospital-based controls, population-based controls. When only the high-quality studies were recruited for meta-analysis, this association was also not found. Publication bias was also tested and not found for *GSTT1*. Our results indicate that the *GSTT1*-null genotype does not predict the RCC susceptibility. The sample size in our meta-analysis was larger than other meta-analyses [43, 48–50].

The average *GSTM1*-null/*GSTT1*-null genotype distribution frequency in patients with RCC is slightly increased. This could indicate that the dual-null genotype, of individuals lacking both *GSTM1* and *GSTT1*, might be associated with the susceptibility of RCC. However, further meta-analysis to detect the risk of the *GSTM1*-null/*GSTT1*-null genotype for RCC susceptibility showed no association between the *GSTM1*-null/*GSTT1*-null genotype and RCC susceptibility in the overall population of Caucasians, compared to hospital-based controls, when only high-quality studies were recruited in the meta-analysis. However, the dual-null genotype was associated with the onset of RCC in Asians, when compared to population-based controls. There was no publication bias for this meta-analysis. As above, the sample size in our meta-analysis was larger than other meta-analyses [48, 50].

The association of the *GSTP1* A/G gene polymorphism with the susceptibility of RCC was also characterized. The average A allele distribution frequency of *GSTP1* in patients with RCC was similar when compared with that in control group, suggesting that there was no association of the *GSTP1* A/G gene polymorphism with RCC susceptibility. We also conducted a meta-analysis and confirmed that the *GSTP1* A/G gene polymorphism is not associated with RCC risk in the overall population of Caucasians and Asians examined, and regardless of whether controls were hospital-based or population-based, and whether high quality studies were solely used. No publication bias was found in this meta-analysis. Furthermore, the sample size in this meta-analysis was notable larger than other meta-analyses [43, 49].

We have also assessed the relationship between *GSTM1* and clinical TNM stages in patients with RCC. The average *GSTM1*-null genotype distribution frequency in stage I + II is slightly lower when compared with that in stage III + IV RCC (I + II/III + IV = 0.85). This might indicate that the *GSTM1*-null genotype is associated with RCC TNM stage. However, meta-analysis of the high-quality studies indicates no association of *GSTM1*-null genotype with clinical TNM stages of RCC is present in the overall population of Caucasians and Asians, compared to hospital-based controls. Interestingly, the *GSTM1*-null genotype is associated with the clinical TNM stages of RCC when the meta-analysis included controls from the population. The sample size of our meta-analysis is notable larger than other meta-analyses [29]. However, more studies are required for confirmation.

The relationship between *GSTT1* and clinical TNM stages of RCC is also assessed. The average *GSTT1*-null genotype distribution frequency in stage I + II is notably lower when compared with that in stage III + IV RCC (I + II/III + IV = 0.76). This might indicate a lack of association of the *GSTT1*-null genotype with clinical TNM stages of RCC in Caucasians and Asians, when compared to population-based controls (Table 4). When only the high-quality studies were included for meta-analysis, this association was also found (Table 4). Interestingly, the *GSTT1*-null genotype is found to be associated with the clinical TNM stages in patients with RCC in the overall population when the meta-analysis includes hospital-based controls. The *GSTT1*-null genotype is also found to be associated with the clinical TNM stages in patients with RCC in the overall population, when compared to hospital-based controls, and in the meta-analysis including high quality studies. Again, the sample size of our meta-analysis is larger than a previous meta-analysis [49]. However, more studies should be performed.

Cheng et al. [50] conducted a meta-analysis that included six investigations for *GSTM1*, six reports for *GSTT1*, and four studies for the dual-null genotype for *GSTM1* and *GSTT1*, and reported that no association was found between the *GSTM1*-null/*GSTT1*-null genotype and RCC susceptibility. The authors also performed a *GSTM1-GSTT1* interaction analysis and indicated that the dual *GSTM1/GSTT1*-null genotype was not significantly associated with the susceptibility of RCC. Liu et al. [51] performed a meta-analysis on eight studies and showed that the *GSTM1*-null genotype was not significantly associated with susceptibility of RCC. Yang et al. [49] conducted a meta-analysis recruited 10 studies of *GSTM1*, 10 reports of *GSTT1*, and five studies of *GSTP1*, and reported that *GSTM1*, *GSTT1* and *GSTP1* gene polymorphisms were not associated with the development of the RCC disease. Jia et al. [43] performed a meta-analysis on 10 studies of *GSTM1*, 10 reports of *GSTT1*, five studies of dual *GSTM1-GSTT1*-null genotype, six studies of *GSTP1*, and concluded that *GSTM1*, *GSTT1*, and *GSTP1* gene polymorphisms were not to be associated with the risk of RCC. Also, *GSTM1-GSTT1* interaction analysis indicated that the dual null genotype for *GSTM1/GSTT1* was notably associated with an increased RCC susceptibility. Huang et al. [48] analyzed eight studies of *GSTM1*, eight studies of *GSTT1*, three studies of *GSTM1* gene polymorphism and clinical TNM stages, and four studies on *GSTM1* and *GSTT1* gene polymorphism and clinical TNM stages, and indicated that *GSTM1* and *GSTT1* gene polymorphisms were not markedly associated with RCC susceptibility in a recessive model. However, comparison of the wild-type genotype versus the dual *GSTM1-GSTT1*-null genotype showed a positive association with the susceptibility of RCC. The authors also identified an association of wild-type *GSTT1* with low RCC TNM stages. A strong association between *GST* genotypes and polymorphism and risk of renal cancer is not there in the total population. The conclusion of all these studies is that *GST* genotypes and polymorphisms cannot be used as biomarkers for early diagnosis.

In this meta-analysis, there are some limitations. First, there was heterogeneity among the recruited studies for the reason that the patients and controls were from different races, and the controls were population-based or hospital-based. Second, geographic origin might affect the relationship between *GSTs* gene polymorphism and RCC susceptibility, and we did not conduct a sub-group analysis. Furthermore, the quality of the recruited articles was different. These factors might prevent us from drawing a more robust conclusion. In addition, although our sample size is larger than prior meta-analyses, but more original studies continue to be needed to draw a more robust conclusion. More well-designed investigations should be conducted in the future.

## Conclusion

The results in this study support that there is an association of the dual *GSTM1-GSTT1*-null genotype with RCC susceptibility in Asians, and there is an association between the *GSTT1*-null genotype and clinical TNM stage of RCC in the overall population. However, more association studies are required to be conducted to further clarify these relationships.

## Additional file

Additional file 1: **Table S1**. Scale for Quality Assessment. (DOC 44 kb)

## Abbreviations

GSH: Glutathione
GST: Glutathione S-transferase
RCC: Renal cell carcinoma
TNM: Tumor node metastasis
